# DNA Tetrahedron Delivery Enhances Doxorubicin-Induced Apoptosis of HT-29 Colon Cancer Cells

**DOI:** 10.1186/s11671-017-2272-9

**Published:** 2017-08-15

**Authors:** Guiyu Zhang, Zhiyong Zhang, Junen Yang

**Affiliations:** 1 Department of Infectious Diseases, Tangshan Gongren Hospital, Tangshan, Hebei 063000 China; 2 Department of Pathology, Tangshan Gongren Hospital, Tangshan, Hebei 063000 China; 3 Department of Cardiology, Tangshan Gongren Hospital, Tangshan, Hebei 063000 China

**Keywords:** DNA tetrahedron, Doxorubicin, Folic acid, Apoptosis, Colon cancer

## Abstract

As a nano-sized drug carrier with the advantage of modifiability and proper biocompatibility, DNA tetrahedron (DNA tetra) delivery is hopeful to enhance the inhibitory efficiency of nontargeted anticancer drugs. In this investigation, doxorubicin (Dox) was assembled to a folic acid-modified DNA tetra via click chemistry to prepare a targeted antitumor agent. Cellular uptake efficiency was measured via fluorescent imaging. Cytotoxicity, inhibition efficiency, and corresponding mechanism on colon cancer cell line HT-29 were evaluated by MTT assay, cell proliferation curve, western blot, and flow cytometry. No cytotoxicity was induced by DNA tetra, but the cellular uptake ratio increased obviously resulting from the DNA tetra-facilitated penetration through cellular membrane. Accordingly, folic acid-DNA tetra-Dox markedly increased the antitumor efficiency with increased apoptosis levels. In details, 100 μM was the effective concentration and a 6-h incubation period was needed for apoptosis induction. In conclusion, nano-sized DNA tetrahedron was a safe and effective delivery system for Dox and correspondingly enhanced the anticancer efficiency.

## Background

Doxorubicin (Dox) is one of the most widely used antineoplastic agent, and numerous clinical studies demonstrated that Dox can strikingly hinder the growth of tumor cells in various cellular growth cycles by inhibiting the synthesis of RNA and DNA [1, 2]. Previous studies suggested that the proliferation of tumor cells were effectively blocked in G1 phase, and the metastasis was also inhibited by Dox at a certain concentration [3]. In addition, effective inhibition can be achieved in a relatively smaller dose when compared with other anticancer drugs [4]. However, Dox usually induced side effects resulted from the lack of specific targeting for tumor cells and nonselective inhibition of DNA and RNA, which seriously limited the clinical applications [5, 6]. Meanwhile, the low cellular intake capacity reduces the accumulation of Dox in tumor cells [7]. Therefore, an efficient delivery system for Dox should be developed to make Dox more specific and effective targeting, more easily to be encapsulated, and of an excellent intake capacity and bio-compatibility.

Nano-sized drug carriers, such as liposome and inorganic nanoparticles, may facilitate Dox penetrating the tumor cell membrane, improving the targeting efficiency [8]. Nonetheless, liposome-based delivery cannot reduce the side effects to normal cells because of the relatively poor targeting; meanwhile, inorganic carriers, such as mesoporous silica nanoparticles, cannot be completely biodegraded in vivo, hampering the process of further drug uptake and bringing potential bio-toxicity. For these functional delivery systems, the complexity of preparation, inhomogenization of nanoparticles structures, and low encapsulation efficiency obstacle the clinical expansions [9–11]. As a nano-sized drug carrier with the excellent performance on drug delivery, DNA-based structures, such as DNA tetrahedron (DNA tetra), may penetrate the membrane via avoiding the incompatibility between electro-negative DNA and plasma membrane [12–17]. Drugs and targeting molecules can be both covalently attached to DNA tetrahedron. Furthermore, easy absorption and biodegradation of DNA tetrahedral sequences avoid long-time retention. Guanine-rich aptamer drugs, such as AS1411, have been successfully delivered into A549 tumor cells and performed as targeting agents and inhibitors [18]. Immune regulatory factors, such as CpG and siRNA, can also be delivered to tumor cells via DNA tetra carrier to regulate immune responses [19]. With the advantages of low-toxicity, proper biocompatibility, and adjustable targeting, DNA tetra showed bio-safety and potentials for Dox delivery.

In this study, functional particles of DNA tetrahedron assembled with Dox as anticancer drug, and folic acid as specific recognition molecules [20], were designed, synthesized, and characterized. The anticancer efficiency was evaluated on colon cancer cells considering significantly upregulated folate receptors on the surface of cell membrane. Specifically, the level of cellular uptake, the degree of Dox-induced apoptosis, and the inhibition on cellular proliferation were measured on HT-29 cell lines.

## Methods

### Regents and Equipment

DNA oligonucleotide chains were purchased from TAKARA in Dalian, China. Dulbecco’s modified Eagle’s medium (DMEM) and fetal bovine serum were purchased from Gibco in NY, USA. Penicillin and streptomycin were purchased from Beyotime biotechnology in Shanghai, China. Folic acid, doxorubicin, 3-(4,5-dimethyl-2-thiazolyl)-2,5-diphenyl-2-*H*-tetrazolium bromide (MTT), and agarose were purchased from Sigma-Aldrich in MO, USA. All the antibodies were purchased from Abcam Company in Shanghai, China. Other reagents were purchased from Sinopharm Chemical Regent Co., Ltd., in Shanghai, China.

UV-Vis spectrophotometer (Thermo Evolution 201) and constant temperature incubator were purchased from Thermo Fisher in the USA; centrifugal machine (GT10-1) was purchased from Beijing Era Beili Centrifuge Co., Ltd.; fluorescence spectrophotometer (UV-1800) was purchased from Shimadzu Corporation. Confocal laser scanning microscopy (Visitech) was purchased from Leica companies; polymerase chain reaction instrument (PCR, T100), protein electrophoresis, and nucleic acid electrophoresis apparatus were purchased from Bio-Rad Company; dynamic light particle size analyzer was purchased from Beckman Company; cell culture plates with 96-well or 24-well were purchased from Dow Corning Corporation. High-performance liquid chromatography (HPLC, Agilent 1200) with C18 column was purchased from Agilent Technologies.

The human colon cancer cell line HT-29 was maintained in DMEM supplemented with 10% fetal bovine serum, 100 U/mL penicillin, and 100 μg/mL streptomycin in a humidified atmosphere containing 95% air and 5% carbon dioxide at 37 °C.

### Synthesis and Purification of DNA Tetrahedron

**Fig. 1:**
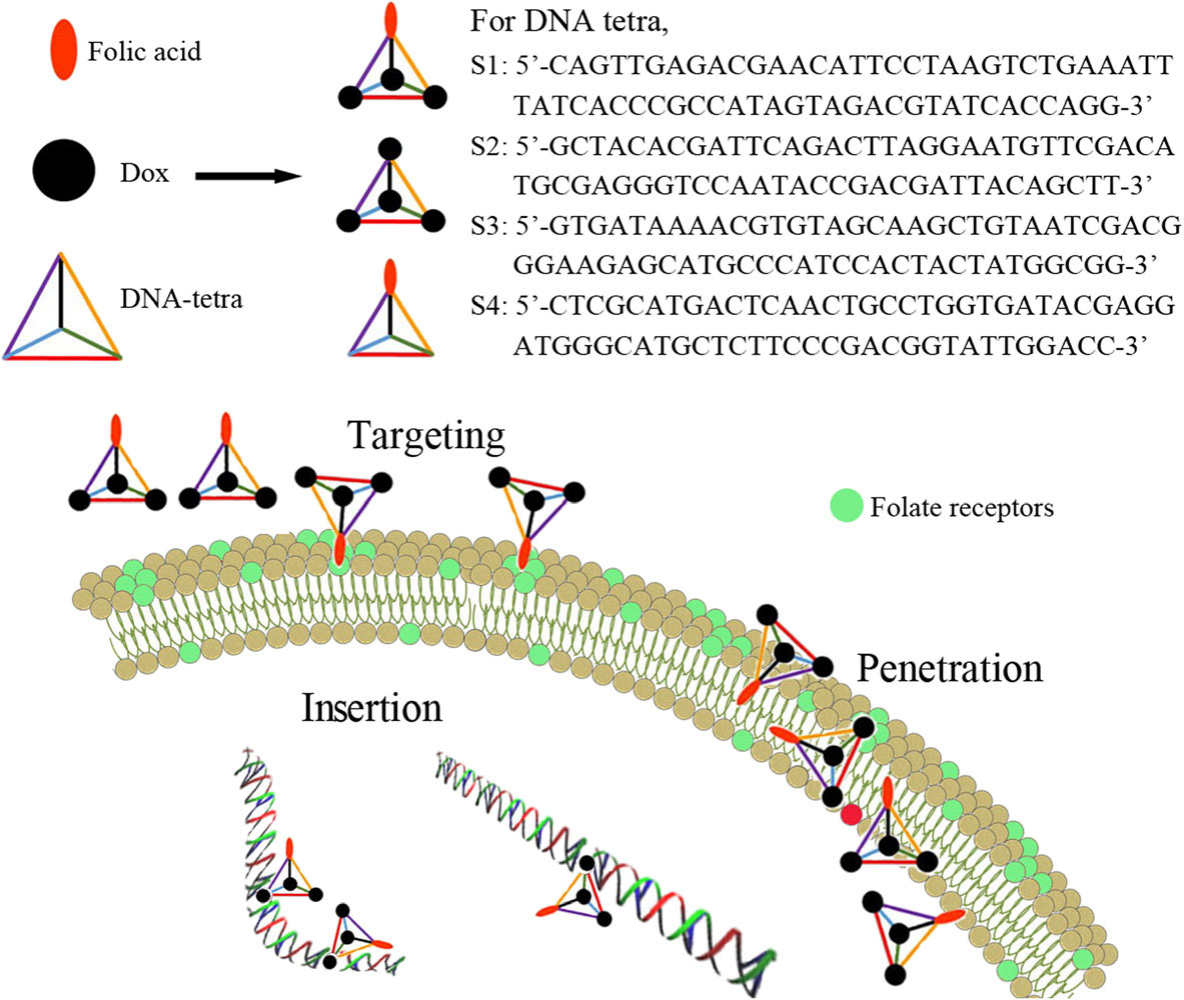
Schematic diagram of folic acid-DNA tetra-Dox, DNA tetra-Dox, and folic acid-DNA tetra. The single-strand DNA sequences of DNA tetrahedron were provided. The process of targeting for tumor cells, penetrating through the cellular membrane, and inserting the DNAs were depicted

In this investigation, synthesis followed the schematic procedures in Fig. 1, and the single-strand DNA (ssDNA) sequences of DNA tetrahedron are provided in Fig. 1 as well. In details, each ssDNA was dissolved in 0.5× TE buffer, and the corresponding optical density (OD) value of DNA was determined by UV spectrophotometer at 260 nm. Additional TE buffer was supplemented to make four chains at the same concentration. The mixing ratio of four ssDNAs was 1:1:1:1 at 1 μM in 100 μL. The reaction was performed in a polymerase chain reaction (PCR) machine with the cycling conditions: 95 °C, 10 min, naturally cooled to 4 °C. All of the single-strand DNA were purified by HPLC with 260 nm as characteristic absorption peak. In HPLC spectrum, the peak time of DNA tetra was faster than that of single strand, and product was collected at the corresponding time point.

### Synthesis and Characterization of Folic Acid-DNA tetra-Dox

Free hydrogen groups of Dox and folic acid were modified with azide group and then coupled with 3′-OH of ssDNA via click chemistry reaction [21]. When adding a different amount of functional group tagged ssDNA, the ratio of functional groups could be stoichiometrically controlled via specific hybridization of side chains. For synthesis of folic acid-DNA tetra, the molar ratio of folic acid and DNA tetrahedron was set as 1:1. For synthesis of DNA tetra-Dox, the molar ratio of Dox and DNA tetrahedron was set as 4:1. For synthesis of folic acid-DNA tetra-Dox, the molar ratio of folic acid, DNA tetrahedron, and Dox was respectively set as 1:1:3. All the synthesis were carried out at micromolar level at 37 °C and then stored at 4 °C [22].

In this study, the series of DNA tetra complexes was characterized by polyacrylamide gel electrophoresis (PAGE) with 8% separation gel (39% Acr-Bis) to check the purity and relative molecular sizes. The samples consisted of the different DNA tetra structures and 6× loading buffer with mixing ratio of 2:1. The samples were stained and analyzed after gel electrophoresis for 90 min at 110 V. To figure out the differences on particle sizes, DNA tetra samples were also scanned by dynamic light scattering (DLS) instruments with dynamic light.

### DNA tetra-Facilitated Cellular Uptake

For the different coupling structures designed in this research, the uptake rates by HT-29 cells were compared to figure out the drug delivery efficiency. Cellular uptake efficiency was evaluated and quantified utilizing the characteristic fluorescence spectrum of Dox, i.e., excitation light at 470 nm and emission light at 590 nm. The HT-29 cells at 2 × 10^5^/mL were seeded in 24-well plates and cultured for 24 h. Cells were incubated with various DNA tetra structures at 10 μM for another 24 h. The medium was then discarded, and the cells were rinsed with PBS three times. For cell fixation, 4% paraformaldehyde was immediately added at room temperature for a 30-min co-incubation, and the cells were rinsed with PBS three times again. At last, the 24-well plates were observed by laser confocal microscope to compare the cellular uptake efficiency based on the light intensity of emission light.

### Cytotoxicity and Anticancer Efficiency

Cytotoxicity and anticancer efficiency were evaluated using MTT assay, where a redox reaction occurs between the MTT in DMSO and intracellular succinate dehydrogenase. HT-29 cells were seeded in 96-well culture plates and cultured for 24 h.

For the cytotoxicity of DNA tetra as drug carrier, medium containing DNA tetra structures at the concentration of 0–100 μM was added for another 24- or 48-h incubation. Then, 100 μL of MTT solution (5 mg/mL) was added to each well, and the mixture was incubated at 37 °C for 4 h. The liquid was then removed, and the cells were lysed and dissolved with 200 μL DMSO. The absorbance of supernatant was measured at 570 nm by Microreader. The non-treated HT-29 sample was deemed as control group.

For the DNA tetrahedron coupled with folic acid or Dox, on the one side, the research focused on the stability, bio-safety, and inhibition efficiency; on the other side, whether the Dox of DNA tetra-Dox has comparable antitumor properties as before or not was also explored. Therefore, cytotoxicity and antitumor efficiency of DNA tetra complexes were evaluated. Complexes respectively at 100 μM were incubated with HT-29 cells. Cell samples were collected every 6 h and then detected by MTT assay to evaluate the effect of incubation period. Particular attentions were paid for the difference on anticancer efficiency resulted from the variations of structures. Furthermore, concentration effects on inhibition of HT-29 after being treated with folic acid-DNA tetra-Dox were evaluated at 0–200 μM via MTT assays.

### Western Blot and Flow Cytometry

To characterize the cellular apoptosis induced by Dox facilitated by DNA tetra delivery, protein samples from treated cells were heated and cracked using pyrolysis liquid, analyzed by 12% SDS-PAGE under the condition of 100 V at constant current to separate samples, and then blotted to 0.22-μm-diameter PVDF membranes for 1 h. The samples were blocked with skimmed milk for 1 h. After being washed for three times with PBST, samples were incubated overnight with rabbit anti-caspase-3. Then, the samples were probed with a goat-anti-rabbit IgG secondary antibody for 1 h and then washed with PBST and imaged via Bio-Rad protein imaging system. Further, GAPDH was chosen as internal reference protein since its stable expression in cells.

Flow cytometry was further utilized to quantify the levels of apoptosis at the selected concentration and time point via MTT assays. HT-29 cells were incubated with inhibitors for a period of time and then measured via quantitative flow cytometry using Annexin V-PI double staining method.

### Cell Proliferation Quantifying

Cell counting method was used to quantify the cell proliferation with time. In details, after being treated with folic acid-DNA tetra-Dox for a period of time at 100 μM, cells were digested by 0.25% trypsin for 30 s, and then, the equal volume of complete medium was added to terminate reaction. Supernatant was discarded after centrifugation in 1000 rpm for 3 min, and cells were re-suspended with complete medium. The number of cells in each detection point was recorded under optical microscope using cell count plates, so as to draw the cellular proliferation curve.

### Statistics Analysis

In this research, significances of differences were determined using Student’s *t* test (two-tailed; two-sample equal variance). *P* < 0.05 means significant differences between different groups.

## Results

### Preparation of Folic Acid-DNA tetra-Dox

**Fig. 2:**
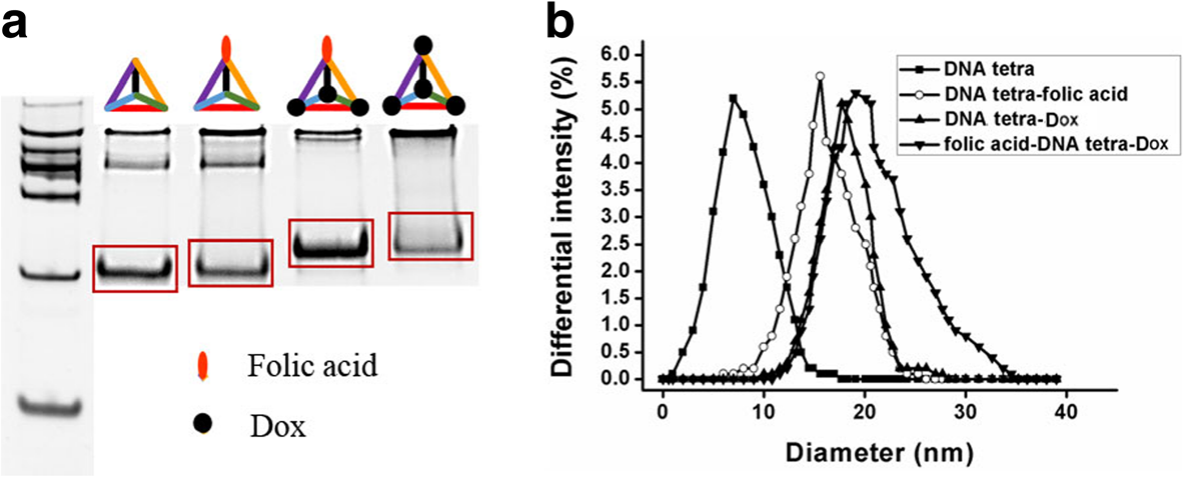
Characterization of DNA tetra with different constituents. **a** Left to right: DNA ladder, DNA tetra, folic acid-DNA tetra, folic acid-DNA tetra-Dox, and DNA tetra-Dox imaged in 8% SDS-PAGE. **b** The size distribution of DNA tetra, folic acid-DNA tetra, folic acid-DNA tetra-Dox, and DNA tetra-Dox detected via dynamic light scattering

In specific synthesis process, Dox was mixed with DNA tetrahedron in different proportions to complete loading and assembling of Dox (*w*_*m*_ = 543.52) and folic acid (*w*_*m*_ = 441.4). As shown in Fig. 2a, due to that Dox and folic acid are monomers with relative low and similar molecular weight, DNA in a tetrahedron and the conjugates with one functional molecule had roughly equivalent molecular weights. However, Dox or folic acid assembling with all the four DNA vertices of the tetrahedron made molecular size of DNA tetra increased obviously. As shown in Fig. 2b, most of DNA tetrahedral monomer size was less than 15 nm. With the increase of coupling groups, the symmetrical structure of DNA tetra was destroyed and the medium of particle sizes of compounds increased significantly. In particular, size expansion of folic acid-DNA tetra-Dox extended the medium of diameter to 20 nm.

### Drug Delivery Efficiency

**Fig. 3:**
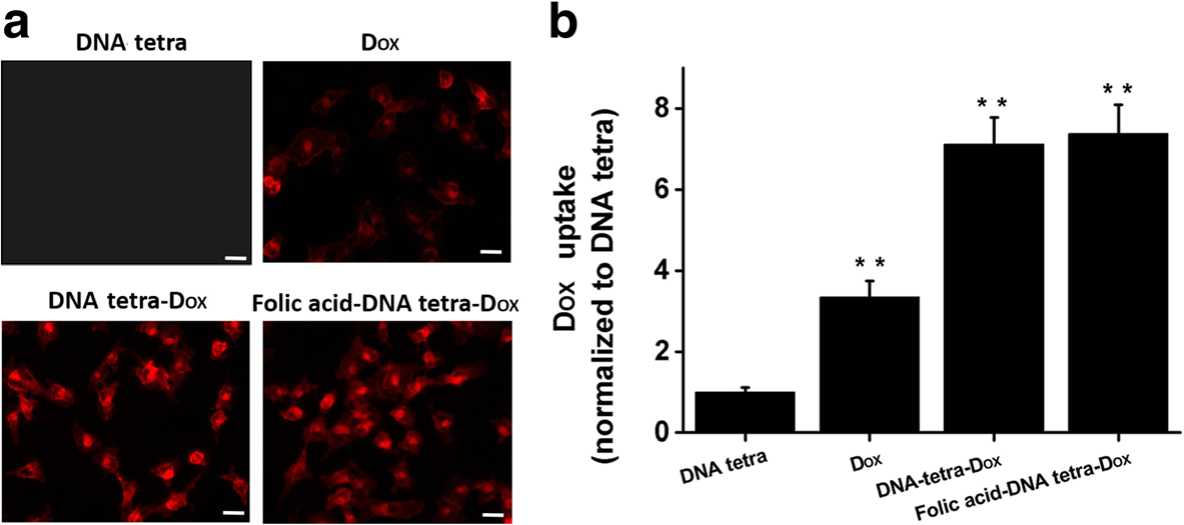
The uptake efficiency of HT-29 cells after being incubated with different complexes for 24 h. **a** Confocal microscopy detection (red is fluorescence excitation light of Dox, scale bar = 10 μm). **b** Intensity of cellular fluorescence. **P < 0.05 when compared with the group of DNA tetra

After being incubated with different DNA tetra structures for 24 h, the intracellular efficiency of Dox was evaluated with fluorescence imaging, where the intracellular red fluorescence is the characteristic fluorescence of Dox; hence, cells incubated with DNA tetra did not exhibit any fluorescent signal as shown in Fig. 3a, and the red signals of cells incubated with folic acid-DNA tetra-Dox and DNA tetra-Dox complexes were obviously higher than that with Dox, meaning the significantly enhanced cellular uptake efficiency of Dox resulting from the DNA tetra facilitated penetration through the membrane, as well as the intracellular stability of the conjugates between Dox and DNA tetra.

The further quantitative analysis of fluorescence intensity (Fig. 3b) evidenced the visual findings, and there was no significant difference between folic acid-DNA tetra-Dox and DNA tetra-Dox complexes (*P* < 0.05). The methodology of co-incubation of drugs and cells obstacles the complete exhibition of targeting ability of folic acid. The relative high concentration confirmed the specific recognition of folate receptors, which is theoretically more obvious in the in vivo applications with the complex circulating system. In brief, the DNA tetra delivery guaranteed the anticancer efficiency of Dox.

### Cytotoxicity and Anticancer Efficiency

**Fig. 4:**
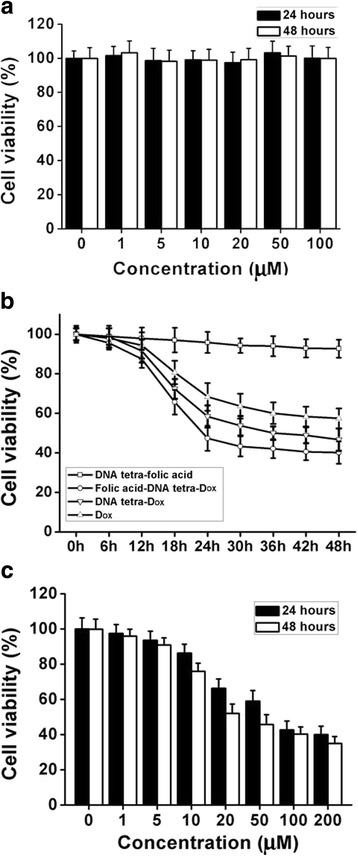
The cytotoxicity and antitumor efficiency of Dox complexes and DNA tetra for HT-29 cells. **a** The viability of HT-29 cells incubated with DNA tetra at different concentrations for 24 or 48 h. **b** The antitumor efficiency of Dox complexes at 100 μM. **c** The viability of HT-29 cells incubated with folic acid-DNA tetra-Dox at different concentrations for 24 or 48 h

First, the viability of HT-29 cells co-incubated with different concentrations of DNA tetra were examined using MTT assay. There were no obvious cytotoxicity of DNA tetra treatment in HT-29 cells at 0–100 μM for 24 and 48 h (Fig. 4a). Hence, the nano-sized DNA tetrahedron provided a bio-safe drug carrier platform.

Secondly, resulting from the difference on Dox delivery efficiency, folic acid-DNA tetra-Dox and DNA tetra-Dox exhibited more significant inhibition efficiency within 48 h (Fig. 4b). Due to the lack of effective anticancer components, DNA tetra-folic acid lead to negligible decrease (< 10%) of viability during the 48-h co-incubation. Due to that Dox cannot objectively penetrate the membrane, Dox-induced decrease of cellular viability was less than the other groups. Particularly, The folic acid-DNA tetra-Dox showed a more earlier and significant decrease at 6 h post incubation, proving that the folic acid targeting help the drugs to locate at the membrane, and make the Dox take effect more timely. By contrast, DNA tetra-Dox began to take effect obviously at 12 h post incubation, demonstrating the importance of targeting groups.

Furthermore, HT-29 cells were treated with folic acid-DNA tetra-Dox at 0–200 μM to detect the effective concentration (Fig. 4c). Cellular viability of HT-29 cells declined rapidly with increasing concentration (0–100 μM) of the complex, but there were no significant differences between the concentration of 100 and 200 μM, indicating a dose-dependent manner of folic acid-DNA tetra-Dox, where 100 μM can be considered as an effective concentration for antitumor effect. In addition, cellular viability changed significantly from 24 to 48 h for the groups of 10, 20, and 50 μM, indicating that the time period of incubation was also a factor in affecting folic acid-DNA tetra-Dox based anticancer efficiency.

### Apoptosis Induced by Folic Acid-DNA tetra-Dox

**Fig. 5:**
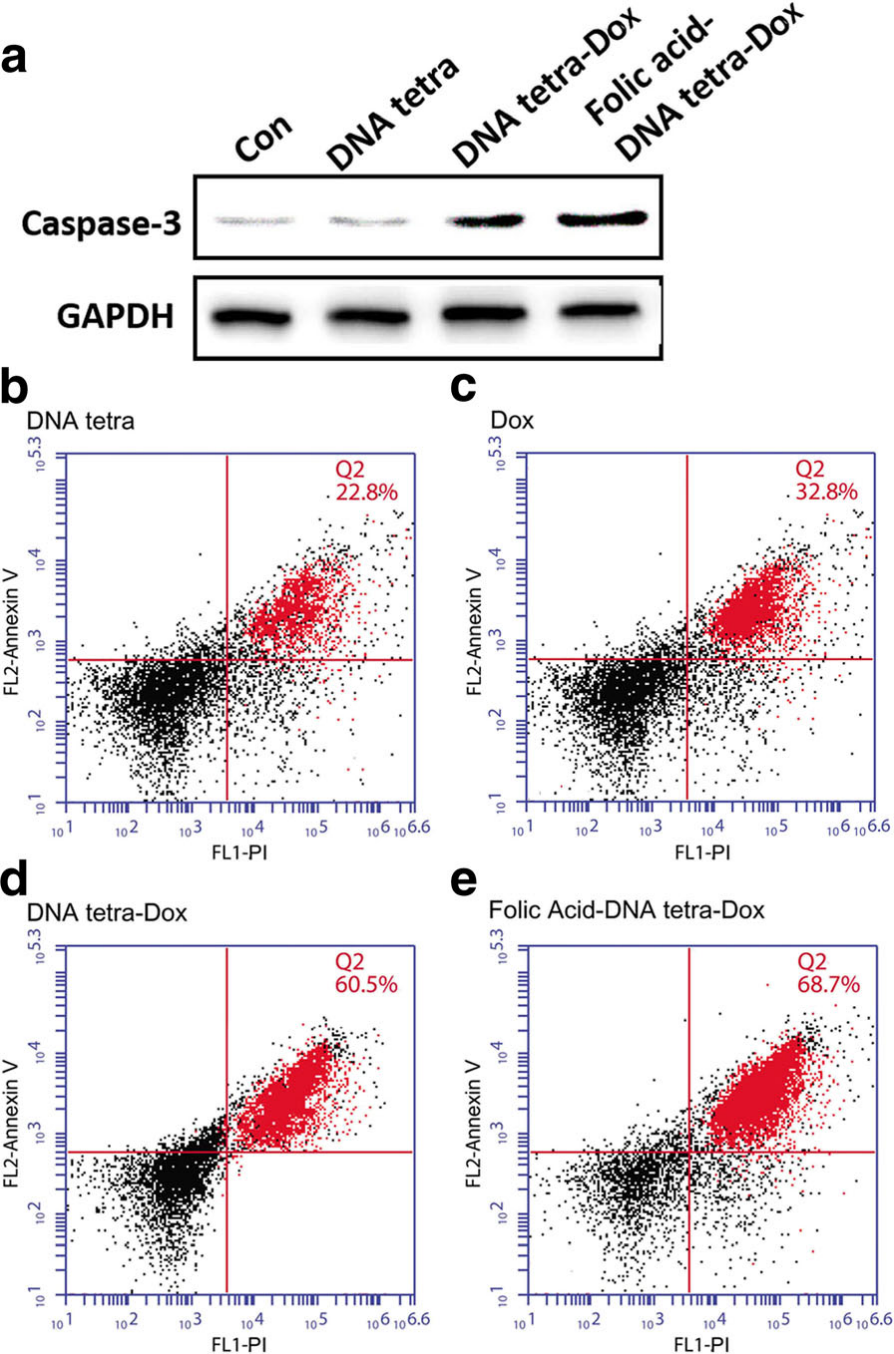
The cellular expression of apoptosis-related caspase-3 after being treated with different complexes (**a**) and the flow cytometry of HT-29 cells after being incubated with DNA tetra, Dox, DNA tetra-Dox, and folic acid-DNA tetra-Dox (**b**–**e**)

Accordingly, based on the western blot assays (Fig. 5a), there were significant increase of the expression of caspase-3 induced by folic acid-DNA tetra-Dox and DNA tetra-Dox, proving that apoptosis was the main way of Dox-induced cell death.

The flow cytometry (Fig. 5b–e) further proved that 6-h incubation with folic acid-DNA tetra-Dox at 100 μM induced 68.7% apoptosis. Meanwhile, the apoptosis levels induced by Dox and DNA tetra-Dox were only 32.8 and 60.5% with the same incubation condition.

### Inhibition on Cell Proliferation

**Fig. 6:**
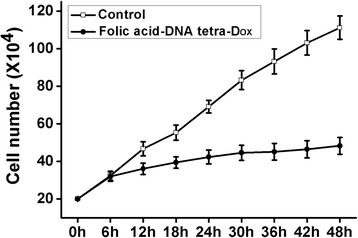
Inhibition of cell proliferation induced by the incubation with folic acid-DNA tetra-Dox at 100 μM for different time periods

Based on the MTT assays, 100 μM was the effective concentration for apoptosis induction and was selected in the cellular proliferation experiment. As shown by the cellular proliferation curve (Fig. 6), due to the time-consuming process of cellular uptake, the inhibition on cell proliferation was not totally shown during the early stage of co-incubation. A significant inhibitory effect was presented after being incubated with folic acid-DNA tetra-Dox for more than 6 h, proving the existence of DNA tetra-enhanced endocytosis. Meanwhile, 6 h is a comparative time period with the effective time period detected in Fig. 4b.

## Discussion

As the common means of DNA modification, click chemistry reaction was of advantages of high reaction efficiency, well-control, and facile operation [21]. Electrophoretogram displayed with a single brand (Fig. 2a), indicating that the products with high purity were obtained by covalent coupling via click chemistry reaction. The fluorescent property of Dox make it trackable in vitro and in vivo; meanwhile, the fluorescence inside the tumor cells indirectly proved the stability of folic acid-DNA tetra-Dox during the process of penetration through the cell membrane. Therefore, the folic acid-DNA tetra-Dox was capable and stable for biomedical applications.

DNA tetra has the capability to deliver drugs into cells. All DNA sequences used in this research were not encoded for any genetic information. Thus, no side effects on both gene expression and cell metabolism were reported in all examinations. Meanwhile, due to the high expression of folate receptor on surface of tumor cells, folic acid is selected as specific targeting molecules of the drug delivery system to enhance the uptake efficiency of DNA tetra complexes. However, with the circumstances of DNA tetra at relatively high concentration, the advantage of folate receptor targeting was not fully reflected in vitro. Accordingly, for in vivo application, the folic acid was potential to enhance the targeting efficiency in the complex circulating system.

Currently, anticancer drugs commonly bring unexpected side effects, so the research on enhancing the tumor cells targeting ability and delivery efficiency has been a hot topic [23–25]. Previous studies showed that capacity of DNA tetrahedron for carrying drugs was based on its good compatibility. DNA tetrahedron is an artificial container of favorable modification and proper biocompatibility. In this study, the successful coupling of Dox and folic acid with DNA tetrahedron achieved satisfying inhibitory effect and lead to obvious apoptosis of HT-29 cell. Owing to that DNA tetra can be modified and coupled with drugs and targeting molecules, the enrichment of local drug concentration in tumor cell was realized. The above results proved the good recognition for tumor cells and corresponding enhanced inhibitory effect via DNA tetra delivery, and this nano-sized drug delivery system can be used more extensively.

## Conclusions

As a newly developed drug delivery strategy, nano-sized DNA structures are of low cost, high stability, and feasibility to synthesize; meantime, it is bio-safe due to the lack of exogenous immune activity. DNA tetrahedron coupling strategy facilitates the targeted delivery of Dox, enhances the anticancer efficiency of Dox on colon cancer cells, and provides a promising inspiration and idea for drug design.
